# Six-Month Predictive Value of Diuretic Resistance Formulas in Discharged Heart Failure Patients after an Acute Decompensation

**DOI:** 10.3390/jcm9092932

**Published:** 2020-09-11

**Authors:** Mauro Feola, Arianna Rossi, Marzia Testa, Cinzia Ferreri, Alberto Palazzuoli, Guido Pastorini, Gaetano Ruocco

**Affiliations:** 1 Cardiology Division, Ospedale Regina Montis Regalis, Mondovi’ ASL CN1, 12084 Cuneo, Italy; marzia.testa@aslcn1.it (M.T.); cinzia.ferreri@aslcn1.it (C.F.); guido.pastorini@aslcn1.it (G.P.); gaetanomaria.ruocco@aslcn1.it (G.R.); 2 School of Geriatry, Universita’ degli Studi Torino, 10124 Torino, Italy; arirossi_ary@hotmail.com; 3 Cardiovascular Diseases Unit, Ospedale Le Scotte Universita’ Siena, 53100 Siena, Italy; palazzuoli2@unisi.it

**Keywords:** NT-proBNP, prognosis, heart failure, diuretic resistance

## Abstract

Background. The diuretic response has been shown to be a robust independent marker of cardiovascular outcomes in acute heart failure patients. The objectives of this clinical research are to analyze two different formulas (diuretic response (DR) or response to diuretic (R-to-D)) in predicting 6-month clinical outcomes. Methods: Consecutive patients discharged alive after an acute decompensated heart failure (ADHF) were enrolled. All patients underwent N-terminal-pro hormone BNP (NT-proBNP) and an echocardiogram together with DR and R-to-D calculation during diuretic administration. Death by any cause, cardiac transplantation and worsening heart failure (HF) requiring readmission to hospital were considered cardiovascular events. Results: 263 patients (62% male, age 78 years) were analyzed at 6-month follow-up. During the follow-up 58 (22.05%) events were scheduled. Patients who experienced CV-event had a worse renal function (*p* = 0.001), a higher NT-proBNP (*p* = 0.001), a lower left ventricular ejection fraction (*p* = 0.01), DR (*p* = 0.02) and R-to-D (*p* = 0.03). Spearman rho’s correlation coefficient showed a strong direct correlation between DR and R to D in all patients (r = 0.93; *p* < 0.001) and both in heart failure with reduced ejection fraction (HFrEF) (r = 0.94; *p* < 0.001) and HF preserved ejection fraction (HFpEF) (r = 0.91; *p* < 0.001). At multivariate analysis, a value of R-to-D <1.69 kg/40 mg, but only <0.67 kg/40 mg for DR were significantly related to poor 6-month outcome (*p* = 0.04 and *p* = 0.05, respectively). Receiver operating characteristic (ROC) curve analyses demonstrated that DR and R-to-D are equivalent in predicting prognosis (area under curve (AUC): 0.39 and 0.40, respectively). Only R-to-D was inversely related to in-hospital stay (r = −0.23; *p* = 0.01). Conclusion: Adding diuresis to DR seemed to provide a better risk assessment in alive HF patients discharged after an acute decompensation.

## 1. Introduction

In heart failure (HF) patients the evaluation of prognosis is currently performed on multiple clinical and laboratory variables, but it remains unpredictable because of sudden hemodynamic deterioration related to cardiac and extracardiac reasons and the lack of consistent universally validated algorithm [1]. The presence of renal dysfunction and arterial hypotension may stratify patients with the worst clinical outcome after discharging for an acute decompensated heart failure episode [2]. A single evaluation of brain natriuretic peptide (BNP) plasma level proved to be a robust risk stratification method being a sensitive diagnostic marker of left ventricular dysfunction [3,4] and predicting cardiovascular events in HF patients [5,6,7,8]. The fluid overload seemed to be crucial for several clinical features which characterize the whole spectrum of congestive heart failure (CHF). Loop diuretics are a cornerstone of CHF therapy, but the question of whether high dosage of diuretics may cause “per se” a worsening clinical prognosis remained unanswered [9]. Diuretic resistance has been widely defined as an impaired sensitivity to diuretics resulting in reduced natriuresis and diuresis limiting the obtaining of euvolemia [10]. In acute decompensation, the development of worsening renal function, called “cardiorenal syndrome”, may be a limitation in achieving an adequate volume removal [11]. If an effective decongestion is not achieved, a modest decline in renal function (calculated by serum creatinine plasma level elevation from to 0.3 to 0.5 mg/dL) has been associated with longer hospitalizations and increases mortality rate [12,13]. Furthermore, other clinical experiences [14,15] concluded that a transient decline in renal function during diuretic therapy in acute decompensation should not be interpreted as an unfavorable event. For instance, the last grade of worsening renal function (<30 days or longer) seemed to influence the mortality risk (hazard ratio (HR) from 0.8 to 3.2) [14] and that the worst prognosis has been observed in those who developed an increase of creatinine plasma level >0.1 mg/dL per day in the first week. Despite the clinical importance of this issue, the definition of “diuretic resistance” has long remained empirical and not quantitative, not measurable. Valente et al. [16], using the measurement of the weight change on Day 4 per 40 mg of furosemide on Days 1–3 (equivalent doses: bumetanide 1 mg; torasemide 20 mg), calculated the “diuretic response” (DR), considered to be the first clinical experience to give a quantitative measurement of diuretic resistance. A worse diuretic response (the lowest quintiles corresponded to the worst prognosis) was independently correlated with 180-day mortality (HR 1.42), 60-day death or renal or cardiovascular rehospitalization (HR 1.34) and 60-day rehospitalization (HR 1.57). These data represented a robust indicator that a blunted loss in weight in CHF patients admitted for acute decompensation described a “diuretic resistance”.

More recently, a formula including diuresis on the fourth day of diuretic treatment, demonstrated to predict cardiovascular outcomes in a single-center clinical experience (R-to-D ≤ 1.2 kg/40 mg furosemide *p* = 0.009, log rank 10.96) [17].

This clinical research aims to verify if the inclusion of diuresis in the formula for diuretic response stratified prognosis in CHF patient treated with loop diuretics during an acute episode of cardiac decompensation (RESEARCH REGISTRY1965) at a short-term follow-up.

## 2. Methods

All consecutive HF subjects discharged alive after an acute episode of cardiac decompensation with fluid overload (clinically or according to water composition) were enrolled in an out-patient clinic follow-up (from January 2017 to December 2019). Patients were classified as having CHF according to the criteria commonly accepted in literature [18], such as the presence of 2 major criteria or 1 major criterion +2 minor criteria according to the Framingham score and a NYHA functional class II, III or IV, due to an exacerbation of symptoms with at least 1 class deterioration. Patients with symptoms of CHF, plasma NT-proBNP > 125 pg/mL and left ventricular ejection fraction (LVEF) < 50% were defined as both in heart failure with reduced ejection fraction (HFrEF). Patients with symptoms of CHF, plasma NT-proBNP > 125 pg/mL, LVEF > 50% and diastolic dysfunction were defined as HF preserved ejection fraction (HFpEF). The presence of inadequate echo images or no adherence to the therapy (a Morisky scale < 1 point) and disagreement with the periodical follow-up were considered exclusion criteria. Eligible patients underwent a clinical examination, a 12-lead electrocardiogram, BNP plasma level determination, body weight measurement at admission and on Day 4 of hospitalization, water composition (on admission and at discharge), 6-min walk test (6MWT), noninvasive cardiac output and a transthoracic echocardiogram within 48 h of hospital discharge. Serum creatinine was checked on clinical stability and glomerular filtration rate (GFR) calculated with the Chronic Kidney Disease-Epidemiology Collaboration (CKD-EPI) equation. The measurement to “diuretic response”, as described by Valente et al. [16], was calculated as follows:

DR = [(W_d4_ − W_baseline_)/Fdose] (1)

where W_d4_ is the weight at day 4 (in kg), W_baseline_ is the weight at baseline, Fdose is the dose of furosemide on days 1–3 (40 mg) (equivalent doses: bumetanide 1 mg; torasemide 20 mg).

In our patient’s population, a new response-to-diuretic (R-to-D) index including both weight changes and diuresis on Day 4 was calculated as follows:

R-to-D = [(W_d4_ − W_baseline_)/Fdose] + log_n_ diuresis (2)

where W_d4_ is the weight at day 4 (in kg), W_baseline_ is the weight at baseline, Fdose is the dose of furosemide on Days 1–3 (40 mg) (equivalent doses: bumetanide 1 mg; torasemide 20 mg) and diuresis refers to the diuresis at Day 4 (in liters).

According to the formula, the log_n_ of a number <1 is a negative number allowing an amplification of the effect of a Day-4 diuresis <1 L/day [17] which was considered one of the criteria for a safe discharge of HF patients after an acute decompensation together with improvement of NYHA class, heart rate < 100 bpm, pulse oximetry in ambient air > 90%, 90 < systolic blood pressure < 120 mmHg [19].

According to the study protocol, CHF outpatients were checked at 3 and 6 months after discharge. In the event of a worsening of the clinical status (worsening dyspnea, body weight increase or edema, cardiac arrhythmias), a clinical control was provided. The long-term medical therapy associated with loop diuretic included: angiotensin converting enzyme inhibitors (ACE) (enalapril, ramipril), angiotensin receptor blockade (valsartan) in the case of enalapril/ramipril intolerance, beta-blockers (bisoprolol), digoxin and spironolactone at low dose. For beta-blockers, ACE and angiotensin receptor blockade, the patients’ maximum tolerated dose was used, after an adequate titration period.

### 2.1. Echocardiography Assessment

Echocardiograms were performed with a Vivid 7 computed sonography system (GE Medical Systems, Waukesha, Wisconsin, USA) according to the recommendations of the American Society of Echocardiography [20]. Two-dimensional apical 2- and 4-chamber views were used for volume measurements; left ventricular ejection fraction (LVEF) was calculated with a modified Simpson’s method using biplane apical (2- and 4-chamber) views. All the echo examinations were performed by expert operators blinded to the results of BNP assay; the intra-observer variability in the evaluation of LVEF was found to be <5%. Echocardiographic measurements including LV end-diastolic diameter and the diastolic thickness of the ventricular septum and the posterior LV wall were determined according to the American Society of Echocardiography recommendations [20]. The tricuspid annular plane systolic excursion (TAPSE) was measured in a four-chamber view by placing the 2D cursor at the tricuspid lateral annulus and measuring the distance of systolic annular RV excursion along a longitudinal line defining the end of systole as the end of the T-wave in the electrocardiogram. Systolic right ventricular (or pulmonary artery) pressure was calculated using the modified Bernoulli equation: PAP = 4 × (tricuspid systolic jet)^2^ +10 mmHg (estimated right atrial pressure).

### 2.2. Blood Collection

At the time of presentation, a blood sample was collected into tubes containing ethylene diaminetetracetic acid and blood was immediately processed. The NT-pro-BNP was performed with a commercially available immunoassay (ADVIA Centaur XPT, Siemens Healthcare Diagnostics, Inc. Tarrytown, NY, USA) on an ADVIA Centaur analyzer according to the established methods.

The assay results were complete in 15 min. Performance characteristics of the test: Assay range 35–35,000 pg/mL; Total CV 9.2–11.4%.

### 2.3. Cardiovascular Events

Clinical outcome was evaluated in terms of death or recurrent heart failure hospitalization over a 6-month follow-up period. A scheduled outpatient visit or phone contact at 90 and 180 days after discharge was provided. Heart failure recurrence was defined as any hospital admission with a primary or secondary diagnosis of volume overload or low output due to pump failure, acute coronary syndrome complicated by heart failure, ventricular arrhythmia associated with left ventricular dysfunction or heart failure related to WRF. All these events were defined through our results as composite outcome in terms of adverse events/cardiac mortality. The study protocol conforms to the ethical guidelines of the 1975 Declaration of Helsinki, it was registered in Research Registry n.1965 and approved by the Ethical Committee of ASLCN1-ASO Santa Croce (ethical approved number 10–18 September 2018). Written informed consent was obtained from all participants prior to the study.

### 2.4. Statistical Analysis

All data were analyzed with intention-to-treat principles. Continuous variables are expressed as median interquartile range (IQR), while discrete variables are presented as counts with percentages (%). The Mann–Whitney U test and χ^2^-test were used as indicated to compare among groups. Spearman rho’s correlation coefficient was employed to evaluate the correlation between R to D formula and DR formula. The receiving operating characteristics (ROC) curve was employed to evaluate outcome prediction by R to D and DR formulas. The Cox regression analysis was used to assess the independent relationship between quartiles of R to D and quartiles of DR for the composite outcome of rehospitalization or death with adjustment for age, gender, hypertension, diabetes mellitus, coronary artery disease (CAD) and chronic kidney disease (CKD). All statistical tests were two-tailed, with *p* value < 0.05 considered significant. All the analyses were performed by using the SPSS 20.0 for Windows (SPSS, IBM, Armonk, NY, USA).

## 3. Results

Two-hundred and sixty-three patients (62% male, age 78 (70–83) years old) were discharged after a new diagnosis of CHF or for acute decompensation in chronic/de novo CHF and were requested to enter the study. All patients were treated with furosemide intravenous for acute decompensation; none was treated with torasemide/bumetanide. None had been treated with thiazide diuretics and 110/205 in the no-event group vs. 32/58 in the event group assumed aldosterone antagonist (*p* = ns). The etiology of HF was interpreted as: 47% ischemic; 11% cardiomyopathy; 20% hypertensive and finally 22% valvular or others. During the 6-month follow-up 58 (22.05%) events were scheduled (21 cardiac deaths, 37 rehospitalization for HF) defining the event group. The remaining 188 patients who did not experience any predefined cardiovascular events formed the no-event group.

Patients who experienced a CV-event had worse renal function (*p* = 0.001), higher pro-BNP (*p* = 0.004), lower DR (*p* = 0.02) and R-to-D (*p* = 0.03) (Table 1); not surprisingly, the furosemide dosage on Day 4 proved to be higher in the event group (*p* = 0.01), while neither the amount of fluid output on Day 4 nor the body weight were significantly different in the two groups (*p* = 0.2 and *p* = 0.56, respectively).

**Figure 1:**
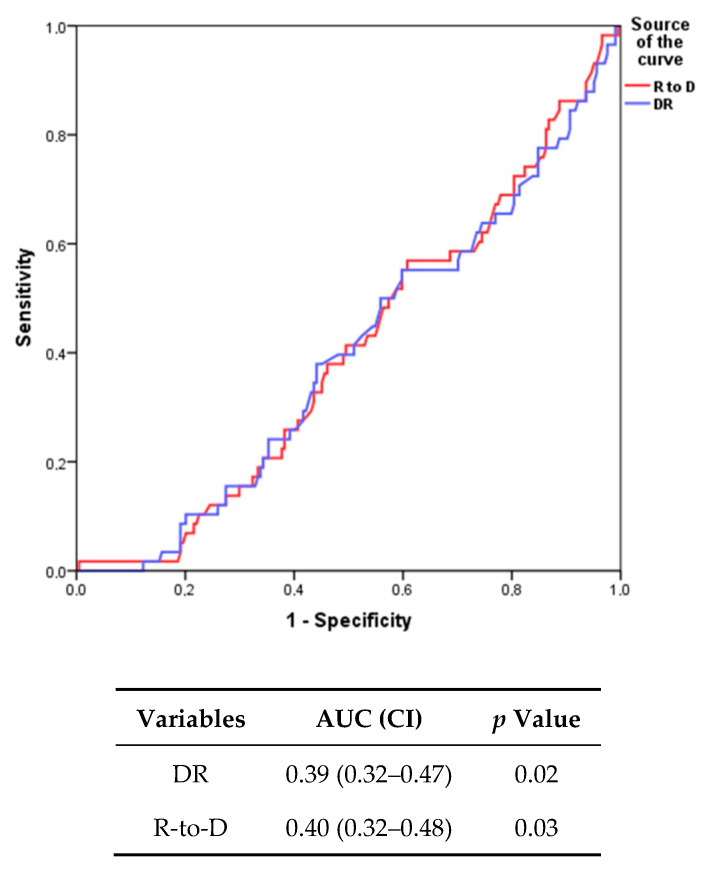
Receiving operating characteristics (ROC) curve analysis comparing diuretic response (DR) and response to diuretic (R-to-D). AUC, area under curve; CI, confidence interval.

In all patients median R to D was 1.69 (0.97–3.28) and median DR was 1.44 (0.67–2.80). Only R-to-D was inversely related to in-hospital stay (r = −0.23; *p* = 0.01). ROC curve analysis showed that both DR (area under curve (AUC): 0.39 (0.32–0.47); *p* = 0.02) and R-to-D (AUC: 0.40 (0.32–0.48); *p* = 0.03) were able to predict poor prognosis at 180 days (Figure 1).

Due to significant findings obtained by ROC curve analysis we divided both DR (Q1: DR < 0.67; Q2: 0.67 ≤ DR < 1.44; Q3: 1.44 ≤ DR < 2.80; Q4: DR ≥ 2.80) and R-to-D (Q1: R-to-D < 0.97; Q2: 0.97≤ R-to-D < 1.69; Q3: 1.69 ≤ R-to-D < 3.28; Q4: R-to-D ≥ 3.28) in quartiles according to median values in all patients. Univariate analysis showed that only Q1 of both DR (HR: 2.56 (1.17–5.63); *p* = 0.02) and R-to-D (HR: 2.53 (1.11–5.67); *p* = 0.03) were significantly related to worse outcome. After adjustment for demographic variables and cardiovascular risk factors, multivariable analysis demonstrated that both Q1 (HR: 2.36 (1.02–5.47); *p* = 0.04) and Q2 (HR: 2.52 (1.04–6.10); *p* = 0.04) of R-to-D were related to poor prognosis; conversely only Q1 of DR (HR: 2.27 (1.01–5.08); *p* = 0.05) was related to poor outcome at 180 days (Table 2).

One-hundred and forty-six patients had HF reduced ejection fraction (HFrEF) and 117 had HF preserved ejection fraction (HFpEF). Patients with HFpEF were younger (76 (68–82) vs. 79 (73–83); *p* = 0.03) and more frequently affected by hypertension (78% vs. 62%; *p* = 0.007) with respect to patients with HFrEF. Conversely, patients with HFrEF were more frequently male (71% vs. 52%; *p* = 0.003) and showed higher rate of diabetes (40% vs. 27%; *p* = 0.04) and CAD (47% vs. 26%; *p* = 0.001) than patients with HFpEF (Table 3).

**Figure 2:**
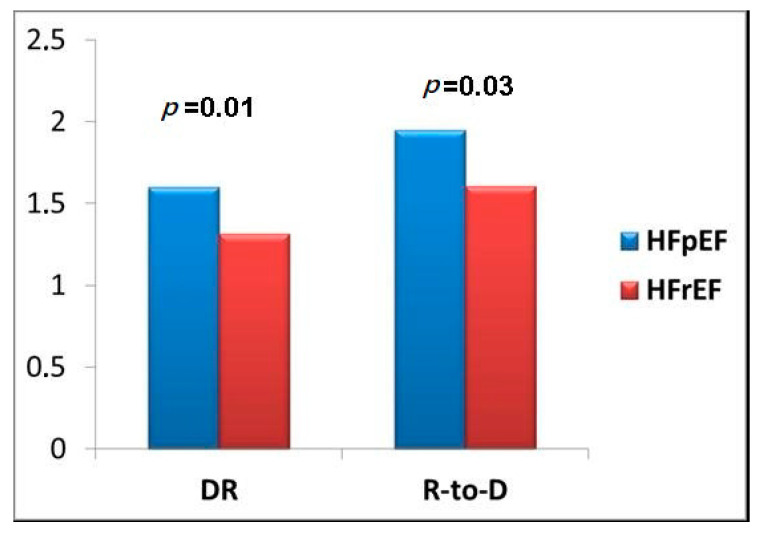
Statistical differences of diuretic response (DR) or response to diuretic (R-to-D) according to the heart failure (HF) classification of reduced/preserved ejection fraction. DR: 1.60 (0.72–3.38) vs. 1315 (0.60–2.24); all patients: 1.44 (0.67–2.80). R to D: 1.95 (1.01–3.75) vs. 1605 (0.94–2.61); all patients: 1.69 (0.97–3.28). HFpEF—heart failure with preserved ejection fraction; HFrEF —heart failure with reduced ejection fraction.

Moreover HFrEF patients showed higher serum levels of creatinine (1.28 (0.94–1.89) vs. 1.02 (0.80–1.25) mg/dL; *p* < 0.001) and hemoglobin (12.9 (11.5–14.2) vs. 11 (10.1–11.9) g/L; *p* < 0.001) and lower values of eGFR (45 (32–61) vs. 53 (45–73) mL/min/m^2^; *p* < 0.001) with respect to HFpEF patients. ProBNP serum levels were significantly higher in HFrEF than HFpEF patients (10,285 (4027–26,078) vs. 3880 (1457–10,686) pg/mL; *p* < 0.001). Comparing diuretic response formulas in our patients divided according to LVEF. There were higher values of both DR (1.60 (0.72–3.38) vs. 1315 (0.60–2.24); *p* = 0.01) and R-to-D (1.95 (1.01–3.75) vs. 1605 (0.94–2.61); *p* = 0.03) in patients with HFpEF with respect to patients with HFrEF (Figure 2).

**Figure 3:**
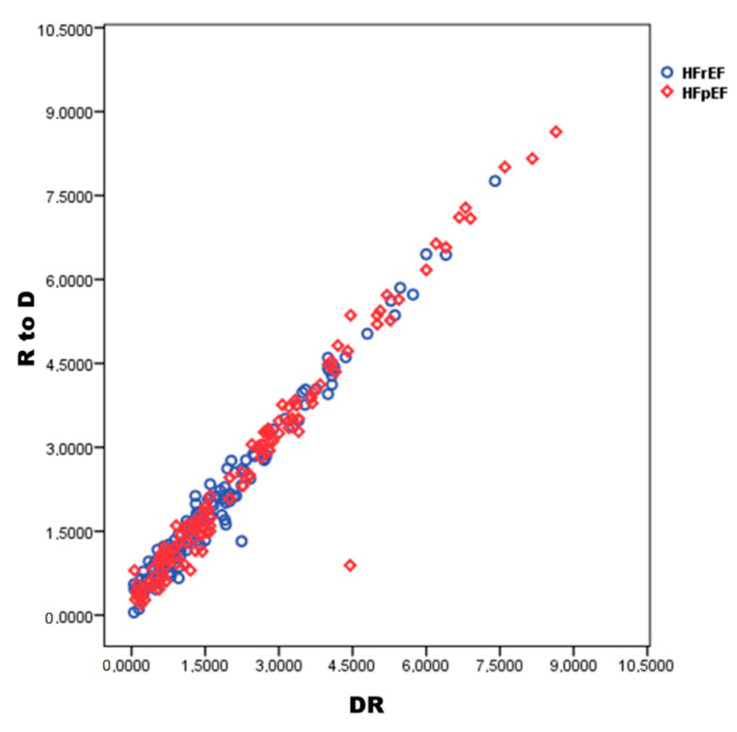
Spearman rho’s correlation between diuretic response (DR) or response to diuretic (R-to-D) in the entire population (263 pts) and both in HFrEF or HFpEF.

Spearman rho’s correlation coefficient showed a strong direct correlation between DR and R-to-D in all patients (r =0.93; *p* < 0.001) and both in HFrEF (r = 0.94; *p* < 0.001) and HFpEF (r = 0.91; *p* < 0.001) (Figure 3).

## 4. Discussion

The most common indicators of diuretic response are net fluid output and changes in body weight. Indeed, body weight reduction does not necessarily imply a consequent decrease in fluid overload [21], but it may be related to an external condition such as sarcopenia cachexia or simple reduction of fluid intake. Recently, a position statement of Heart Failure Association from ESC underlined the utilization of diuretics towards stepped pharmacological diuretic strategies based on diuretic response and common adverse effects (electrolyte disturbances and worsening renal function) [22].

This single-center clinical experience demonstrated that a formula including diuresis together with weight loss during loop diuretic therapy in acute decompensation HF patients seemed to satisfactorily define the clinical outcome after discharge. In CHF patients coming from the PROTECT Trial, Valente et al. [16] calculated the DR as the weight loss per a unitary dose of furosemide (40 mg). A poor diuretic response was independently correlated to low systolic blood pressure, high blood urea nitrogen and predicted 1- and 6-month mortality and cardiovascular hospitalization [16]. Nevertheless, the diuretic responsiveness (diuretic efficiency) has been proposed by calculating the amount of urine produced by the dose of administered loop diuretics [23]. Testani et al. [23] analyzed whether the net fluid output produced per 40 mg furosemide equivalent, may be predictive of clinical outcome in two different populations (coming from the Penn database or from the ESCAPE Trial) admitted for acute decompensation. Dividing into “low” and “high” diuretic efficiency the HF population, they observed that the low diuretic efficiency was correlated to a worsened survival even after adjusting for the diuretic dosage, fluid output and worsening renal function. Unfortunately, despite a persistent increased volume of diuresis after starting a diuretic therapy, a reduction of renal sodium output emerged from the analysis of hypotonic urine diminishing the final objective of the diuretic therapy (to ride the body of excessive sodium). This could explain the interest in dosing the urinary sodium content [24] or only a spot urine sample 1–2 h following loop diuretic administration [25].

In our clinical experience, DR and R-to-D depicted an equivalent ROC curve analysis (AUC 0.39 and 0.40, respectively), although the formula R-to-D, through the inclusion of log_n_ of diuresis on Day 4 to weight loss, proved to better stratify clinical prognosis in alive HF patients discharged after an acute decompensation demonstrating that the lower quartiles (<1.69 kg/40 mg) were significantly correlated to the adverse 6-month prognosis after discharge (*p* = 0.04). In fact, HF patients in whom the R-to-D proved to be <1.69 kg/40 mg had an HR of 2.5 for developing an events in the follow-up, while for DR (not including the diuresis in the formula) the HR of 2.2 emerged only in the most diuretic resistance quartiles (<0.67 kg/40 mg) being not sensitive in case of moderate-light degree of diuretic resistance. Moreover, at univariate analysis, only R-to-D was inversely related to in-hospital stay (r = −0.23; *p* = 0.01). Elderly patients with chronic HF represent most subjects (70%) admitted to the hospitals for acute cardiac decompensation; the length of hospitalization usually lasts >2 weeks in geriatric wards and readmission is frequent [26]. Advanced HF patients constitute a challenge for cardiologists owing their high mortality and rehospitalization rate [27,28]. Therefore, a strategy for stratify planning a tailored clinical follow-up in those patients seems to be mandatory.

The analysis of diuretic resistance in HFrEF and HFpEF demonstrated as DR and R-to-D were significantly higher in HFpEF in comparison to HFrEF (*p* = 0.01 and *p* = 0.03, respectively). This result seemed to be correlated to the worst renal function of HFrEF patients in comparison with HFpEF (*p* = 0.001 for creatinine and eGFR). The diuretic response has been demonstrated to be strictly correlated to renal impairment [16,23] and it has been shown that HFpEF had a better renal function in comparison to HFrEF [29]. In our clinical experience we demonstrated as the response to loop diuretic in HFrEF patients, measured with DR or R-to-D, was impaired in comparison to HFpEF, adding to our knowledge of the differences between the two different HF syndromes. Indeed, diuretic resistance in HErEF together with older age, comorbidities and worse renal function may play a significant role in explaining the unfavorable post-discharge prognosis in comparison to HFpEF patients. Therefore, HFrEF and HFpEF patients discharged alive after an acute decompensated heart failure (ADHF) with an R-to-D in the lower quartiles should be considered at higher risk of rehospitalization or death.

## 5. Conclusions

In the growing number of parameters used to predict adverse prognosis in HF patients, the diuretic resistance should be considered sensitive and inexpensive. Although adding the computation of diuresis to DR gained similar AUC, the R-to-D formula proved to be more accurate in predicting 6-month adverse events in less severe degree of diuretic resistance (lower quartiles) than DR. Only R-to-D was inversely related to in-hospital stay (r = −0.23; *p* = 0.01). Finally, HFrEF patients seemed to respond worst to loop diuretic according to an impaired renal function in comparison to HFpEF subjects.

## 6. Limitations

We did not evaluate renal function by using specific biomarkers, and we cannot assert whether WRF was related to the progressive deterioration specific to HF patients or effective tubular function impairment. A serial analysis of renal function trajectories after discharge is lacking and it may be useful to better understand underlying mechanism of kidney deterioration and diuresis reduction.

## Figures and Tables

**Table 1:** Differences between main variables in event group (180 days rehospitalization or death) vs. no-event group.

Variables	No-Event Group (205 pts)	Event Group (58 pts)	*p* Value
Age	77 (70–82)	79 (76–85)	0.01
Gender Male (%)	63	64	0.88
Sodium (MEq/L)	140 (138–143)	140 (136–142)	0.04
Creatinine (mg/dL)	1.06 (0.82–1.45)	1.53 (1.04–2.05)	<0.001
GFR mL/min/1.73 mq	52 (40–68)	39 (28–50)	<0.001
Hb (gr/L)	12 (10.5–13.7)	12.6 (11.2–13.5)	0.22
RDW	14.8 (13.8–16)	15.9 (14.7–17.7)	<0.001
Pro-BNP (pg/mL)	5210 (1851–14,901)	12,922 (7074–27,769)	<0.001
LVEF (%)	47 (35–55)	38 (26–50)	0.001
LVESD (mm)	37 (30–46)	45 (35–57)	0.008
LVEDD (mm)	51 (46–60)	59 (48–66)	0.04
TAPSE (mm)	17 (15–20)	16 (15–20)	0.32
PASP (mmHg)	37 (30–45)	45 (35–58)	0.003
Length of hospital stay (days)	10 (7–14)	10 (7–15)	0.35
Baseline body weight (kg)	73.9 (63–89.3)	70.9 (56.8–89.9)	0.30
Body weight at Day 4 (kg)	72.4 (60.4–87.8)	70.9 (58–87.4)	0.77
Diuresis on Day 4 (mL/die)	1800 (1300–2600)	1900 (1400–2800)	0.44
Diuretic dosage on Day 4 (mg/die)	60 (50–100)	100 (50–237)	0.005
R-to-D (kg/40 mg furosemide)	1.74 (1.02–3.48)	1.53 (0.77–2.49)	0.03
Diuretic response (kg/40 mg furosemide)	1.50 (0.75–3.20)	1.29 (0.53–2.17)	0.02
Diabetes mellitus N (%)	30	50	0.004
Coronary artery disease N (%)	39	33	0.38
Hypertension (%)	65	81	0.03
CKD (%)	66	83	0.01
AF (%)	34	53	0.02

GFR—glomerular filtration rate; Hb—hemoglobin level; RDW—red dispersion width; pro-BNP—brain natriuretic peptide; LVEF—left ventricular ejection fraction; LVESD—left ventricular end-systolic diameter; LVEDD—left ventricular diastolic diameter; TAPSE—tricuspid annular plane systolic excursion; PASP—pulmonary artery systolic pressure; R-to-D—response to diuretic; CKD—chronic renal disease; AF—atrial fibrillation.

**Table 2:** Univariable and multivariable analysis for the composite endpoint of death from all causes (ACM) or heart failure (HF) rehospitalization according to quartiles of R to D (A) and quartiles of DR (B).

**(A) 180-Day HF Rehospitalization or Death**				
	**Univariate Analysis**		**Multivariate Analysis ^1^**	
**Variable**	**HR (95% CI)**	***p *** **Value**	**HR (95% CI)**	***p *** **Value**
Q1 R to D (R to D < 0.97 kg/40 mg)	2.53 (1.11–5.67)	0.03	2.36 (1.02–5.47)	0.04
Q2 R to D (0.97 ≤ R to D < 1.69 kg/40 mg)	2.09 (0.88–4.93)	0.09	2.52 (1.04–6.10)	0.04
Q3 R to D (1.69 ≤ R to D < 3.28 kg/40 mg)	2.26 (0.97–5.29)	0.06	2.35 (0.99–5.55)	0.06
Q4 R to D (R to D ≥ 3.28 kg/40 mg)	Reference		Reference	
**(B) 180-Day HF Rehospitalization or Death**				
Q1 DR (DR < 0.67 kg/40 mg)	2.56 (1.17–5.63)	0.02	2.27 (1.01–5.08)	0.05
Q2 DR (0.67 ≤ DR < 1.44 kg/40 mg)	1.54 (0.66–3.61)	0.32	1.81 (0.76–4.31)	0.18
Q3 DR (1.44 ≤ DR < 2.80 kg/40 mg)	2.13 (0.94–4.81)	0.07	2.23 (0.97–5.12)	0.06
Q4 DR (DR ≥ 2.80 kg/40 mg)	Reference		Reference	

Quartiles R to D: Q1. Abbreviations: CI—confidence interval; DR—diuretic response; HR—hazard ratio. **^1^** adjusted for age, gender and hypertension, diabetes, CAD and CKD.

**Table 3:** Baseline characteristics of patients according left ventricular ejection fraction (LVEF).

Characteristics	All Patients	HFrEF	HFpEF	*p* Value
	*n* = 263	*n* = 146	*n* = 117	
Age (years)	78 (70–83)	79 (73–83)	76 (68–82)	0.03
Gender Male (%)	62	71	52	0.003
Comorbidities (%):				
CAD	38	47	26	0.001
Diabetes	34	40	27	0.04
Hypertension	69	62	78	0.007
AF	38	41	35	0.42
CKD	69	74	64	0.08
Laboratory variables				
Creatinine (mg/dL)	1.13 (0.85–1.61)	1.28 (0.94–1.89)	1.02 (0.80–1.25)	<0.001
eGFR (mL/min/m^2^)	50 (37–66)	45 (32–61)	53 (45–73)	<0.001
Hemoglobin (g/L)	12.1 (10.7–13.7)	12.9 (11.5–14.2)	11 (10.1–11.9)	<0.001
RDW	14.9 (14–16.4)	15.3 (14.2–16.5)	14.6 (13.6–16)	0.02
NTproBNP (pg/mL)	7851 (2623–17,989)	10,285 (4027–26,078)	3880 (1457–10,686)	<0.001
Echocardiography:				
EDD (mm)	53 (46–60)	60 (51–65)	48 (41–53)	<0.001
ESD (mm)	39 (31–47)	46 (39–55)	32 (28–39)	<0.001
PASP (mmHg)	40 (30–50)	39 (30–50)	40 (31–50)	0.49
TAPSE (mm)	17 (15–20)	16 (15–20)	18 (15–20)	0.31

AF—fibrillation; BNP—b-type natriuretic peptide; CKD—chronic kidney disease; EDD—end diastolic diameter; EDS—end systolic diameter; eGFR—estimated glomerular filtration rate; HFpEF—heart failure with preserved ejection fraction; HFrEF —heart failure with reduced ejection fraction; LVEF—Left ventricle ejection fraction; PASP—pulmonary artery systolic pressure; RDW—red cell distribution width; TAPSE—tricuspid annular plane systolic excursion.
