# Correction: Feola, M., et al. Six-Month Predictive Value of Diuretic Resistance Formulas in Discharged Heart Failure Patients after an Acute Decompensation. *J. Clin. Med.* 2020, *9*, 2932

**DOI:** 10.3390/jcm10030531

**Published:** 2021-02-02

**Authors:** Mauro Feola, Arianna Rossi, Marzia Testa, Cinzia Ferreri, Alberto Palazzuoli, Guido Pastorini, Gaetano Ruocco

**Affiliations:** 1 Cardiology Division, Ospedale Regina Montis Regalis, Mondovi’ ASL CN1, 12084 Cuneo, Italy; marzia.testa@aslcn1.it (M.T.); cinzia.ferreri@aslcn1.it (C.F.); guido.pastorini@aslcn1.it (G.P.); gaetanomaria.ruocco@aslcn1.it (G.R.); 2 School of Geriatry, Universita’ degli Studi Torino, 10124 Torino, Italy; arirossi_ary@hotmail.com; 3 Cardiovascular Diseases Unit, Ospedale Le Scotte Universita’ Siena, 53100 Siena, Italy; palazzuoli2@unisi.it

**Text Correction**

There was an error in the original article [1]. "(a Morinsky scale<1 point)" has been changed to "(medication adherence was assessed using a self-reported measure)".

A correction has been made to *2. Methods Section*, *Paragraph 1*:

All consecutive HF subjects discharged alive after an acute episode of cardiac decompensation with fluid overload (clinically or according to water composition) were enrolled in an out-patient clinic follow-up (from January 2017 to December 2019). Patients were classified as having CHF according to the criteria commonly accepted in literature [18], such as the presence of 2 major criteria or 1 major criterion +2 minor criteria according to the Framingham score and a NYHA functional class II, III or IV, due to an exacerbation of symptoms with at least 1 class deterioration. Patients with symptoms of CHF, plasma NT-proBNP > 125 pg/mL and left ventricular ejection fraction (LVEF) < 50% were defined as both in heart failure with reduced ejection fraction (HFrEF). Patients with symptoms of CHF, plasma NT-proBNP > 125 pg/mL, LVEF > 50% and diastolic dysfunction were defined as HF preserved ejection fraction (HFpEF). The presence of inadequate echo images or no adherence to the therapy (medication adherence was assessed using a self-reported measure) and disagreement with the periodical follow-up were considered exclusion criteria. Eligible patients underwent a clinical examination, a 12-lead electrocardiogram, BNP plasma level determination, body weight measurement at admission and on Day 4 of hospitalization, water composition (on admission and at discharge), 6-min walk test (6MWT), noninvasive cardiac output and a transthoracic echocardiogram within 48 h of hospital discharge. Serum creatinine was checked on clinical stability and glomerular filtration rate (GFR) calculated with the Chronic Kidney Disease-Epidemiology Collaboration (CKD-EPI) equation. The measurement to “diuretic response”, as described by Valente et al. [16], was calculated as follows:

DR = [(W_d4_ − W_baseline_)/Fdose] (1)

where W_d4_ is the weight at day 4 (in kg)_,_ W_baseline_ is the weight at baseline, Fdose is the dose of furosemide on days 1–3 (40 mg) (equivalent doses: bumetanide 1 mg; torasemide 20 mg).

The authors apologize for any inconvenience caused and state that the scientific conclusions are unaffected. The original article has been updated.

**Editorial Note**

Following a request by the license holders of the scale measuring therapeutic adherence referred to in the article, the authors and journal published this correction. The issues have been previously described [2].

The Committee on Publication Ethics (COPE) has noted this type of behavior that is stated to hold “authors to ransom”, and recommends to emphasize that “this is not good for the advancement of scientific knowledge or in the public interest” [3].

## References

[1] Feola M., Rossi A., Testa M., Ferreri C., Palazzuoli A., Pastorini G., Ruocco G. (2020). Six-Month Predictive Value of Diuretic Resistance Formulas in Discharged Heart Failure Patients after an Acute Decompensation. J. Clin. Med..

[2] https://www.sciencemag.org/news/2017/09/pay-or-retract-survey-creators-demands-money-rile-some-health-researchers.

[3] https://publicationethics.org/case/licence-published-scale.

